# 超高效液相色谱-串联质谱法检测婴幼儿辅助食品中多种兽药残留

**DOI:** 10.3724/SP.J.1123.2022.03039

**Published:** 2022-12-08

**Authors:** Ruilian LUO, Zhengshuang WU, Chiqiong LIANG, Liting LUO

**Affiliations:** 1.佛山市食品药品检验检测中心, 广东 佛山 528051; 1. Foshan Center for Food and Drug Control, Foshan 528051, China; 2.佛山市第五人民医院, 广东 佛山 528200; 2. The 5th People’s Hospital of Foshan City, Foshan 528200, China

**Keywords:** 固相萃取净化, 超高效液相色谱-串联质谱, 多兽药残留, 婴幼儿辅助食品, solid-phase extraction purification, ultra performance liquid chromatography-tandem mass spectrometry (UPLC-MS/MS), multi-veterinary drug residues, complementary foods for infants and young children

## Abstract

生产婴幼儿辅助食品的原辅料中常常含有鱼类、肉类、肝类等动物性组织,存在兽药残留的风险,为了更加全面地对这类产品进行安全监管,研究并开发了同时检测婴幼儿辅助食品中6大类(喹诺酮类、磺胺类、大环内酯类、硝基咪唑类、氯霉素类和抗病毒类)50种抗生素和抗病毒类兽药残留的超高效液相色谱-三重四极杆质谱法(UPLC-MS/MS)。样品采用酸化乙腈超声提取,提取液经新型的脂质增强型Captiva EMR-Lipid固相萃取柱净化,浓缩复溶后采用流动相乙腈和0.1%(v/v)甲酸水溶液,经C18柱梯度洗脱分离,电喷雾多反应监测(MRM)模式检测,基质匹配外标法定量。结果显示,该方法50种兽药在0.5~50 μg/L范围内线性关系较好,相关系数均不低于0.995,方法检出限为0.03~0.70 μg/kg,定量限为0.09~2.33 μg/kg。50种化合物在不同基质中,添加5和50 μg/kg两个加标水平进行试验,平均回收率为64.37%~119.3%,相对标准偏差均小于15%。将该方法应用于14份国产和6份进口的婴幼儿辅助食品检测,结果显示,1份进口肉类婴幼儿辅助食品中检出磺胺喹噁啉、磺胺二甲嘧啶和替米考星。该方法简单快速,灵敏度和准确度高,样品量消耗少,适用于婴幼儿辅助食品中多种兽药残留的检测。

## Abstract (en)

Special attention must be paid to children and infants because health issues stemming from a poor immunologic system and low weight make them a vulnerable risk group. Complementary foods for infants and young children, which are a class of special dietary foods, are essential transitional foods for infants from lactation to adaptation to ordinary food. Some complementary foods for infants and young children are of animal origin such as fish, meat, and the liver, which may contain veterinary drug residues. Veterinary drugs are usually small-molecular-weight chemicals that are essential for treating infections, increasing production, and improving animal husbandry. However, abuse of these substances can provoke transfer to the food chain, leading to negative consequences for humans, especially infants and young children. For a more comprehensive safety supervision of infant supplementary foods, a method based on ultra performance liquid chromatography-tandem mass spectrometry (UPLC-MS/MS) was developed and used for the determination of 50 antibiotics and antivirals (grouped into six categories: fluoroquinolones, sulfonamides, macrolides, nitroimidazoles, chloramphenicols, and antivirals) in complementary foods for infants and young children. The matrix of complementary foods for infants and young children is complex and contains a large number of proteins and lipids, which poses a serious challenge for sample pretreatment. The Captiva EMR-Lipid solid-phase extraction (SPE) column is a new type of product that allows for selective and efficient lipid/matrix removal without negatively affecting the recovery of the analyte. In this study, samples were extracted with acidified acetonitrile and then purified using a Captiva EMR-Lipid SPE column. The target analytes were separated on a BEH C18 column by gradient elution using acetonitrile and water containing 0.1% (v/v) formic acid as the mobile phases. MS detection was performed with an electrospray source in the positive and negative modes in the multireaction monitoring (MRM) mode. The ion spray voltages were set at 5500 V and -4500 V in the positive and negative modes, respectively. The source temperature for both the ionization modes was set to 500 ℃. Instrumental parameters such as collision energy and declustering potential were optimized. The samples were quantified using the external standard method with matrix calibration curves to reduce the influence of the matrix effect on the quantitative results.

The results showed that the 50 veterinary drug residues had good linear relationships in the range of 0.5 to 50 μg/L, with correlation coefficients higher than 0.995. The limits of detection (LODs) and quantification (LOQs) were in the range of 0.03-0.70 μg/kg and 0.09-2.33 μg/kg, respectively. The average recoveries for all the compounds under different matrices ranged from 64.37% to 119.3% at spiked levels of 5 μg/kg and 50 μg/kg, with relative standard deviations of less than 15%. Compared to QuEChERS, this method has a better purification effect. The recoveries of the 50 veterinary drugs extracted by this method were also much higher than those in the case of QuEChERS. This method was applied to the detection of 14 domestic and six imported infant supplementary foods. Sulfaquinoxaline, sulfamethazine, and tilmicosin were detected in one imported meat-based baby food. With its simple operation, high sensitivity and accuracy, and low sample quantity consumption, this method is suitable for the determination of multiveterinary drug residues in complementary foods for infants and young children. This study provides an effective analysis method for risk monitoring and troubleshooting of complementary foods for infants and young children, which is of great significance in ensuring the healthy growth of the next generation.

婴幼儿作为一类最特殊、最敏感、最容易受到伤害的群体,其食品安全问题尤其需要得到更多的关注。曾经发生的多起“毒奶粉”事件给人们敲响了食品安全的警钟,这些年我国也在不断地加强对婴幼儿配方乳粉的质量安全监管,出台了一系列最严格的管理政策^[1]^。婴幼儿辅助食品作为一类特殊的膳食食品,是婴幼儿从断乳期到适应普通食物的必备过渡期食品^[2]^,其安全性同样应该得到重视。“亨氏”生产的营养米粉因添加猪肝粉导致铅超标的事件,反映出了婴幼儿辅助食品生产的原料乱象,产品质量存在安全隐患。为加强监督管理,卫生部发布了《食品安全国家标准 婴幼儿谷类辅助食品》^[3]^和《食品安全国家标准 婴幼儿罐装辅助食品》^[4]^,国家食药监总局也先后发布了《婴幼儿辅助食品生产许可审查细则》(2017版)和《关于落实婴幼儿辅助食品生产许可审查细则 严格生产许可工作的通知》^[5,6]^。世界各国和组织如国际食品法典委员会(Codex Alimentarius Commission, CAC)^[7]^、欧盟^[8]^、澳新食品标准局^[9]^、美国^[10]^、日本^[11]^,为保证婴幼儿辅助食品的质量,同样制定了一些标准和相关法规。然而,除CAC规定抗生素和激素不得检出外,其他标准和法规均只规定了婴幼儿辅助食品中污染物、部分农药残留和真菌毒素限量,并未对抗生素和抗病毒等兽药残留制定限量。这意味着各国对婴幼儿辅助食品中的兽药残留是“零容忍”的^[12]^。兽药在畜牧业中常被用于治疗疾病、提高生长和饲养效率。然而,兽药的滥用会使部分药物残留在供人食用的动物源性食品中,从而给食品安全带来威胁。生产婴幼儿辅助食品的原辅料中常常含有鱼类、肉类、肝类等动物性组织,因此存在兽药残留的风险。而且鉴于国外婴幼儿辅食品种丰富,特别是含肉食品,海关总署尚且没有出台相关的法规来统一规范进口^[13]^。我国目前也还没有发布针对婴幼儿辅食多兽药残留的相关检测标准,使得这类产品的监管工作存在盲点和难点。为此,有必要开发一种快速、简便、灵敏度高、检出限尽可能低的测定婴幼儿辅助食品中多兽药残留的方法。

现有的测定多兽药残留的标准方法^[14,15]^和研究^[16⇓⇓⇓⇓⇓⇓-23]^大多应用超高效液相色谱-串联质谱法(UPLC-MS/MS),这是因为其具有分析效率高、灵敏度高等优势。但是,研究对象多为水产品、畜禽肉、鸡蛋、牛奶和奶粉等,针对婴幼儿辅助食品中多兽药残留的研究相对较少。如Aguilera-Luiz等^[12]^采用QuEChERS法检测了肉类婴儿食品和配方奶粉中29种兽药残留,发现部分产品存在少量兽药残留;Jia等^[24]^采用离子阱高分辨质谱同时筛查了婴幼儿食品中333种农药和兽药残留,检出部分阳性产品;Gómez-Pérez等^[25]^同样采用离子阱高分辨质谱分析了添加肉类、鱼类和蔬菜类的婴儿食品中的农兽药残留,所检测的样品均未发现农兽药残留。不足的是,这些研究多采用高分辨质谱筛查,定量结果不够准确,而且仪器昂贵,不利于推广。

婴幼儿辅助食品基质复杂,包含大量蛋白质和脂类,为样品前处理带来了严峻挑战。Captiva EMR-Lipid固相萃取柱是一种新型的脂质增强型去除产品,其空间排阻和疏水性作用可以选择性地去除脂类而不吸附目标化合物,具有良好的净化效果,且不影响分析物的回收率。本研究采用Captiva EMR-Lipid固相萃取柱作为净化手段,对动物源性食品中常有的药物在婴幼儿辅助食品中的残留同时进行了筛查和定量分析,包括喹诺酮类、磺胺类、大环内酯类、硝基咪唑类、抗病毒类和氯霉素类共50种兽药。该方法简单快速、准确可靠,样品量消耗少,为婴幼儿辅食风险监测和隐患排查提供了行之有效的分析方法,对保障下一代的健康成长具有重要意义。

## 1 实验部分

### 1.1 材料与试剂

50种兽药标准品,纯度均大于95%,购自Dr. Ehrenstorfer公司;甲酸、乙腈、甲醇均为色谱纯,购于上海CNW公司;Captiva EMR-Lipid固相萃取柱(3 mL/300 mg)购于安捷伦科技有限公司。

### 1.2 仪器与设备

Triple Quard 5500+超高效液相色谱-三重四极杆质谱仪(UPLC-MS/MS),美国SCIEX公司;Milli-Q超纯水机,德国Merck公司;MV5多通道平行浓缩仪,美国LabTech公司;电子天平;旋涡混合器;超声波清洗器;离心机。

### 1.3 实验方法

#### 1.3.1 标准溶液配制

准确称取50种分析物标准品各约1.0 mg(以单体计)于10 mL容量瓶中,用甲醇溶解,得到质量浓度约为100.0 mg/L的标准储备液,置于-18 ℃下保存。准确吸取上述各储备液适量,用乙腈定容至10 mL,配制成质量浓度为1.0 mg/L的混合标准溶液,于4 ℃下冷藏保存。

基质标准工作溶液的制备:用乙腈将混合标准溶液逐步稀释至质量浓度为5、10、50、100、500 μg/L的系列标准中间溶液。取阴性样品按样品前处理的方法提取,得到空白基质溶液。分别精确吸取各质量浓度的标准中间溶液100 μL于2 mL空白基质溶液中,氮气吹干,以10%(v/v)乙腈水溶液定容至1 mL,即得质量浓度分别为0.5、1、5、10、50 μg/L的系列基质标准工作溶液。

#### 1.3.2 样品前处理

称取1 g样品于50 mL离心管中,加入2 mL水于旋涡混合器上充分混匀(若样品含水量较高,可省略此步),加入4 mL 5%(v/v)甲酸乙腈溶液,置于超声波清洗器中超声20 min, 8000 r/min离心5 min,上清液移至10 mL离心管中,残渣按上述步骤重复提取1次。合并上述2次提取液,用水定容至10 mL,混匀后取2.5 mL过Captiva EMR-Lipid固相萃取柱,取2 mL净化液在40 ℃下N_2_吹干。残渣用10%乙腈水溶液溶解并定容至1 mL后过0.22 μm有机滤膜,滤液用于UPLC-MS/MS测定。

#### 1.3.3 UPLC-MS/MS检测条件

UPLC条件 色谱柱为ACQUITY UPLC^®^ BEH C18(50 mm×2.1 mm, 1.7 μm,购自Waters公司);柱温:35 ℃;进样体积:5 μL;流动相:A为0.1%(v/v)甲酸水溶液,B为色谱纯乙腈;流速:0.2 mL/min。梯度洗脱程序:0~1.0 min, 10%B; 1.0~5.5 min, 10%B~75%B; 5.5~5.7 min, 75%B~90%B; 5.7~6.5 min, 90%B; 6.5~6.7 min, 90%B~10%B; 6.7~8.0 min, 10%B。

质谱参数 电喷雾电离(ESI),正/负离子自动切换模式;多反应监测(MRM);气帘气(CUR)241 kPa,碰撞气(CAD)621 kPa,离子源温度(TEM)500 ℃,离子源气压(GS1, GS2)均为345 kPa,正/负离子喷雾电压(IS)分别为5500 V和-4500 V,正/负离子碰撞室入口电压(EP)分别为8 V和-10 V,碰撞室出口电压(CXP)分别为15 V和-14 V。

## 2 结果与讨论

### 2.1 质谱条件的优化

在化合物优化模式下,采用注射器直接进样,分别在正离子和负离子模式下对母离子和子离子进行扫描,确定定量和定性离子对,在MRM模式下对去簇电压(DP)、碰撞能(CE)、碰撞室入口电压和碰撞室出口电压等参数进行了优化,表1列出了主要的优化参数。50种兽药混合标准溶液的总离子流图如图1所示,可以看出,50种分析物均在5.5 min内被洗脱出来,达到了快速检测的目的。

**表1:** 50种分析物的保留时间及主要质谱参数

Type	Compound	t_R_/min	Qualitative ions (m/z)	Declustering potential/V	Collision energies/eV
Fluoroquinolones	orbiflixacin (奥比沙星)	3.41	396.1/352.1^*^, 396.1/295.1	150	27, 34
	pipemidic acid (吡哌酸)	2.36	304.2/217.2^*^, 304.2/189.1	150	32, 42
	danofloxacin (丹诺沙星)	3.23	358.1/340.1^*^, 358.1/283.1	140	35, 34
	oxolinic acid (噁喹酸)	4.06	262.1/244.1^*^, 262.1/216.1	130	29, 40
	enrofloxacin (恩诺沙星)	3.33	360.2/316.2^*^, 360.2/245.1	130	28, 39
	flumequine (氟甲喹)	4.76	262.1/244.1^*^, 262.1/202.1	130	30, 44
	fleroxacin (氟罗沙星)	3.07	370.1/326.1^*^, 370.1/269.1	140	27, 38
	ciprofloxacin (环丙沙星)	3.12	332.1/314.1^*^, 332.1/231.1	150	32, 50
	lomefloxacin (洛美沙星)	3.25	352.1/265.1^*^, 352.1/308.1	150	33, 25
	marbofloxacin (麻保沙星)	2.94	363.1/72.1^*^, 363.1/345.1	125	40, 30
	nalidixic acid (萘啶酸)	4.66	233.1/187.1^*^, 233.1/215.1	110	36, 23
	norfloxacin (诺氟沙星)	3.04	320.1/276.2^*^, 320.1/302.1	140	25, 33
	pefloxacin (培氟沙星)	3.10	334.1/316.1^*^, 334.1/290.1	130	33, 26
	sarafloxacin (沙拉沙星)	3.59	386.1/342.1^*^, 386.1/299.1	150	27, 40
	difloxacin (双氟沙星)	3.64	400.1/356.1^*^, 400.1/299.1	140	29, 42
	sparfloxacin (司帕沙星)	3.62	393.2/349.2^*^, 393.2/292.1	155	29, 36
	cinoxacin (思诺沙星)	3.80	263.1/217.1^*^, 263.1/245.1	140	34, 27
	ofloxacin (氧氟沙星)	3.07	362.1/318.1^*^, 362.1/261.1	130	27, 39
	enoxacin (依诺沙星)	2.94	321.1/303.1^*^, 321.1/232.1	130	31, 49
Sulfonamides	sulfapyridine (磺胺吡啶)	2.39	250.1/156.0^*^, 250.1/184.1	90	25, 23
	sulfameter (磺胺对甲氧嘧啶)	3.20	281.1/156.0^*^, 281.1/126.1	80	25, 40
	sulfamethazine (磺胺二甲嘧啶)	3.10	279.1/186.1^*^, 279.1/124.1	80	22, 40
	sulfisoxazole (磺胺二甲异噁唑)	3.98	268.1/156.0^*^, 268.1/113.1	110	22, 25
	sulfisomidine (磺胺二甲异嘧啶)	3.10	279.1/156.0^*^, 279.1/186.1	120	28, 26
	sulfamethoxazole (磺胺甲噁唑)	3.82	254.1/156.0^*^, 254.1/108.1	80	24, 37
	sulfamerazine (磺胺甲基嘧啶)	2.63	265.1/156.0^*^, 265.1/172.0	70	25, 25
	sulfamethizole (磺胺甲噻二唑)	3.17	271.1/156.0^*^, 271.1/108.0	100	22, 37
	sulfamethoxypyridazine (磺胺甲氧哒嗪)	3.20	281.1/156.0^*^, 281.1/126.1	110	26, 35
	sulfadimethoxine (磺胺间二甲氧嘧啶)	4.27	311.1/156.1^*^, 311.1/108.1	120	30, 40
	sulfamonomethoxine (磺胺间甲氧嘧啶)	3.51	281.1/156.1^*^, 281.1/126.1	80	22, 39
	sulfaquinoxaline (磺胺喹噁啉)	4.27	301.1/156.0^*^, 301.1/108.0	80	21, 40
	sulfadoxin (磺胺邻二甲氧嘧啶)	3.78	311.1/156.1^*^, 311.1/108.0	100	26, 36
	sulfachloropyridazine (磺胺氯哒嗪)	3.62	285.1/156.0^*^, 285.1/108.0	110	24, 37
	sulfadiazine (磺胺嘧啶)	1.85	251.1/156.0^*^, 251.1/108.1	70	24, 37
	sulfathiazole (磺胺噻唑)	2.24	256.0/156.0^*^, 256.0/108.1	110	23, 35
Macrolides	erythromycin (红霉素)	4.58	734.4/158.2^*^, 734.4/576.4	110	40, 27
	tilmicosin (替米考星)	4.27	869.5/174.2^*^, 869.5/696.5	110	57, 55
Nitroimidazoles	metronidazole (甲硝唑)	1.40	172.1/128.0^*^, 172.1/82.0	85	21, 35
	metronidazole-OH (羟基甲硝唑)	1.20	188.0/123.1^*^, 188.0/126.0	85	20, 23
	dimetridazole (地美硝唑)	1.78	142.1/96.0^*^, 142.1/81.0	85	22, 36
	hydroxydimetridazole (羟甲基甲硝咪唑)	1.47	158.0/140.1^*^, 158.0/55.0	85	18, 32
	ronidazole (洛硝哒唑)	1.82	201.0/140.1^*^, 201.0/55.1	80	18, 32
	ipronidazole (异丙硝唑)	3.90	170.1/124.0^*^, 170.1/109.1	90	24, 35
	ipronidazole-OH (羟基异丙硝唑)	3.17	186.1/168.0^*^, 186.1/122.0	85	20, 29
Antiviral	amantadine (金刚烷胺)	3.08	152.2/135.1^*^, 152.2/93.1	90	25, 39
	rimantadine (金刚乙胺)	4.08	180.2/163.2^*^, 180.2/121.1	90	23, 34
Chloramphenicols	florfenicol amine (氟苯尼考胺)	0.91	248.2/230.2^*^, 248.2/130.2	75	19, 35
	chloramphenicol (氯霉素)	4.11	321.0/152.0^*^, 321.0/257.0	-85	-25, -16
	florfenicol (氟苯尼考)	3.87	356.0/336.0^*^, 356.0/185.0	-85	-14, -27
	thiamphenicol (甲砜霉素)	3.07	354.0/185.0^*^, 354.0/290.0	-95	-30, -18

*Quantitative ion pair.

**图1:**
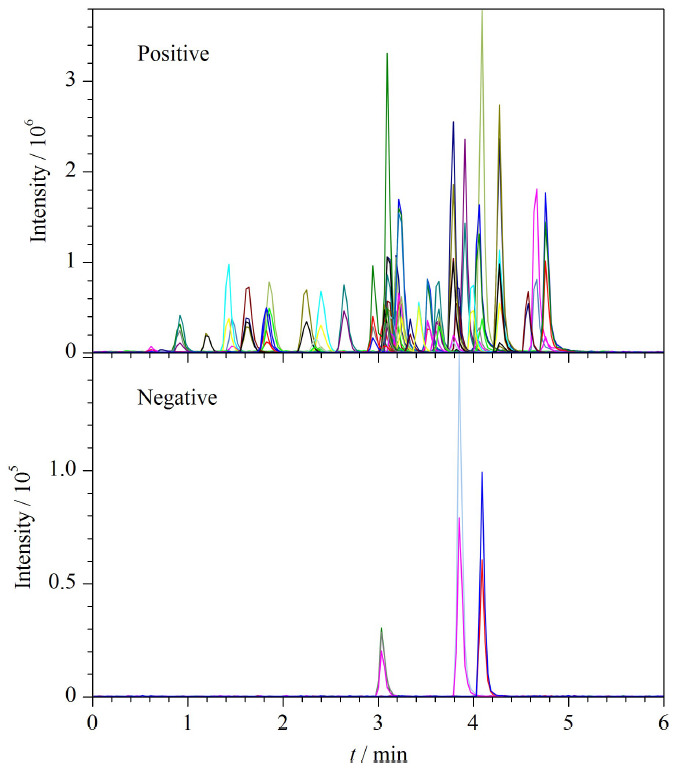
50种分析物混合标准溶液的总离子流图

### 2.2 前处理条件的优化

#### 2.2.1 提取溶剂的选择

根据所发布的食品检测标准和相关文献,食品中兽药提取所使用的有机溶剂通常为甲醇、乙腈、乙酸乙酯、丙酮等。鉴于乙腈对大多数药物的提取效果较好,且具有沉淀蛋白质的能力,本工作选取乙腈为考察对象。另外,本工作所研究的兽药大多为碱性化合物,在碱性条件下较易发生开环反应,因此本方法研究了50种分析物在酸性条件下的提取效果。以婴幼儿米粉基质为例,对比了乙腈中分别加入0.1%、0.5%、1%、5%甲酸时6大类兽药的回收率(见表2)。结果显示,随着甲酸添加量增多,大部分化合物的回收率都显著提升,特别是磺胺类药物,当甲酸浓度较低时,回收率不足60%。当甲酸浓度增加到5%时,50种分析物的回收率均稳定在70%~115%之间。综合考虑,以5%(v/v)甲酸乙腈作为提取溶剂。

**表2:** 不同酸性条件下6大类兽药的回收率

Compound type	Recoveries under different acidic conditions/%			
	0.1%	0.5%	1%	5%
Fluoroquinolones	62.58-81.36	64.67-90.27	82.42-113.3	82.77-114.8
Sulfonamides	39.14-53.82	51.92-70.05	65.36-94.07	73.77-114.4
Macrolides	55.82-71.64	67.67-78.83	76.98-96.74	80.81-94.75
Nitroimidazoles	63.13-86.54	79.24-97.55	87.88-112.9	95.50-113.7
Antiviral	67.33-89.85	84.09-95.55	83.70-96.96	90.46-101.2
Chloramphenicols	87.73-103.4	89.61-110.7	98.78-109.0	92.37-110.3

#### 2.2.2 净化方法的选择

QuEChERS法^[12,18,25]^是目前使用最多的食品中多兽药残留检测的样品前处理方法,因其简单、快速高效而被大多数研究者青睐。本工作以婴幼儿米粉基质为例,对比了QuEChERS^[12]^和Captiva EMR-Lipid固相萃取柱的净化效果。

图2的实验结果表明,采用QuEChERS净化,50种兽药的回收率明显偏低,回收率不到80%,部分药物回收率还不足60%,可能是该法除杂效果不理想,基质抑制效应严重;也可能是吸附剂吸附了部分化合物,导致回收率偏低。而使用Captiva EMR-Lipid固相萃取柱的效果更好,各种分析物的回收率均在70%~115%的范围内。

**图2:**
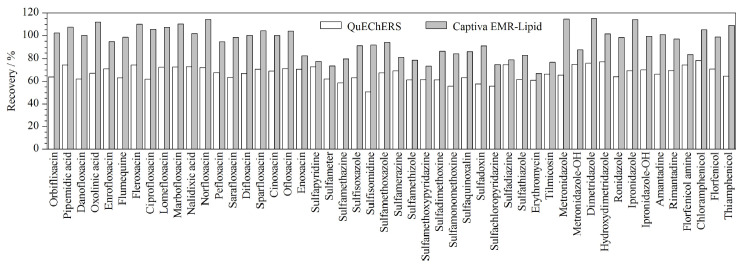
不同净化方法下50种分析物的回收率

#### 2.2.3 基质效应(ME)

为保证定量结果的准确性,基质效应是必须考察的一个重要因素。依据文献^[16,24]^, ME=[基质匹配标准曲线的斜率/纯溶剂标准曲线的斜率-1]×100%,当|ME|为0~20%时,说明存在弱基质效应;当|ME|为20%~50%时,则存在中等基质效应;若|ME|大于50%,表明存在强基质效应。图3列出了50种药物在婴幼儿米粉中的基质效应。结果显示,喹诺酮类既有弱基质抑制效应又有弱基质增强效应,其中达氟沙星存在中等基质增强效应;磺胺类药物大部分存在中等基质抑制效应,少数存在弱基质抑制效应;硝基咪唑类药物存在弱基质抑制效应;大环内酯类和抗病毒类存在弱基质增强效应,氯霉素类除氟苯尼考胺有中等基质增强效应外,其余表现为弱基质增强效应。因此,本实验采用基质匹配标准曲线来校正,以降低基质效应对定量结果的影响。

**图3:**
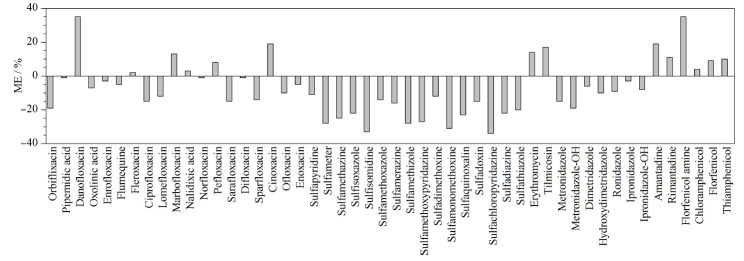
50种兽药的基质效应

### 2.3 方法学评价

#### 2.3.1 标准曲线、检出限和定量限

采用基质匹配外标校正的方法绘制标准曲线,纵坐标(*Y*)为各待测兽药定量离子的色谱峰面积,横坐标(*X*)为相应的质量浓度(μg/L),结果列于表3。结果表明,在0.5~50 μg/L范围内,50种药物均呈现出良好的线性关系,相关系数(*r*)均不低于0.995。

**表3:** 50种兽药的线性关系、LOD和LOQ

Compound	Linear equation	r	LOD/(μg/kg)	LOQ/(μg/kg)
Orbiflixacin	Y=197779X+3757	0.9996	0.22	0.75
Pipemidic acid	Y=87903X-6529	0.9992	0.46	1.53
Danofloxacin	Y=188157X+53899	0.9963	0.20	0.67
Oxolinic acid	Y=435877X+192489	0.9951	0.15	0.49
Enrofloxacin	Y=132617X-15113	0.9990	0.18	0.58
Flumequine	Y=522215X+176642	0.9967	0.09	0.30
Fleroxacin	Y=176090X+10428	0.9993	0.36	1.21
Ciprofloxacin	Y=107427X+55346	0.9954	0.46	1.54
Lomefloxacin	Y=132101X+9999	0.9993	0.13	0.44
Marbofloxacin	Y=60345X+3338	0.9992	0.36	1.19
Nalidixic acid	Y=298336X+65419	0.9980	0.16	0.53
Norfloxacin	Y=51835X-4201	0.9991	0.30	1.02
Pefloxacin	Y=237291X+105448	0.9957	0.19	0.65
Sarafloxacin	Y=87118X+6292	0.9982	0.20	0.66
Difloxacin	Y=128580X-4878	0.9987	0.30	1.00
Sparfloxacin	Y=185686X+4122	0.9997	0.09	0.29
Cinoxacin	Y=36291X+15880	0.9983	0.70	2.33
Ofloxacin	Y=175488X+19008	0.9992	0.23	0.77
Enoxacin	Y=285481X-9663	0.9982	0.13	0.44
Sulfapyridine	Y=265262X+94227	0.9988	0.24	0.80
Sulfameter	Y=428447X+538403	0.9960	0.23	0.77
Sulfamethazine	Y=234091X+246581	0.9951	0.14	0.48
Sulfisoxazole	Y=218544X+130177	0.9962	0.16	0.54
Sulfisomidine	Y=101459X+97429	0.9956	0.11	0.35
Sulfamethoxazole	Y=207321X+132074	0.9958	0.18	0.60
Sulfamerazine	Y=263730X+72356	0.9995	0.21	0.70
Sulfamethizole	Y=184920X+247013	0.9956	0.34	1.14
Sulfamethoxypyridazine	Y=384016X+525753	0.9954	0.03	0.11
Sulfadimethoxine	Y=525803X+461027	0.9970	0.07	0.24
Sulfamonomethoxine	Y=191571X+104637	0.9964	0.17	0.57
Sulfaquinoxalin	Y=199160X+310288	0.9955	0.15	0.51
Sulfadoxin	Y=538404X+448887	0.9958	0.21	0.70
Sulfachloropyridazine	Y=160264X+204651	0.9963	0.11	0.35
Sulfadiazine	Y=288292X+127803	0.9991	0.19	0.63
Sulfathiazole	Y=274049X+123238	0.9955	0.13	0.42
Erythromycin	Y=175371X+61139	0.9973	0.06	0.21
Tilmicosin	Y=26547X-8005	0.9953	0.26	0.87
Compound	Linear equation	r	LOD/(μg/kg)	LOQ/(μg/kg)
Metronidazole	Y=360232X+61918	0.9976	0.03	0.09
Metronidazole-OH	Y=83637X+11760	0.9989	0.22	0.75
Dimetridazole	Y=211275X+13791	0.9994	0.09	0.31
Hydroxydimetridazole	Y=141242X+5589	0.9986	0.26	0.87
Ronidazole	Y=185802X+65378	0.9973	0.31	1.03
Ipronidazole	Y=428540X+40596	0.9981	0.22	0.73
Ipronidazole-OH	Y=364488X+19279	0.9984	0.24	0.79
Amantadine	Y=902516X+181596	0.9981	0.52	1.73
Rimantadine	Y=103634X+256309	0.9961	0.07	0.25
Florfenicol amine	Y=140548X+43509	0.9954	0.09	0.31
Chloramphenicol	Y=30477X+576	0.9990	0.36	1.21
Florfenicol	Y=45215X+8278	0.9980	0.17	0.58
Thiamphenicol	Y=10223X+186	0.9980	0.33	1.09

*Y*: peak area; *X*: mass concentration, μg/L.

本实验在阴性婴幼儿辅助食品基质中添加低浓度的混合标准溶液,按1.3.2节的方法处理后,采用1.3.3节的条件进行检测。根据各化合物的特征色谱峰的信噪比(*S/N*)=3和*S/N*=10时对应的加标水平确定检出限(LOD)和定量限(LOQ),得到婴幼儿辅助食品中50种兽药的LOD为0.03~0.70 μg/kg,LOQ为0.09~2.33 μg/kg。50种药物在婴幼儿米粉基质中的线性关系、LOD和LOQ见表3。

#### 2.3.2 回收率与精密度

以不含目标化合物的婴幼儿辅助食品(米粉、肉泥、鱼泥)为基质,加入一定量的混合标准溶液,使目标化合物在样品中的含量分别为5和50 μg/kg,按1.3.2节方法处理,每个加标水平平行测定6次,以相对应的基质标准曲线定量。结果(见表4)表明,在高低两个加标水平下,各分析物在3种基质中的平均回收率为64.37%~119.3%,方法的RSD小于15%,能够满足兽药残留分析的要求。

**表4:** 50种兽药在3种基质中的平均回收率及相对标准偏差(*n*=6)

Compound	Spiked/ (μg/kg)	Mean recoveries/%				RSDs/%		
		Rice powder	Meat paste	Fish paste		Rice powder	Meat paste	Fish paste
Orbiflixacin	5	87.09	111.7	95.45		9.2	5.1	6.7
	50	90.40	90.36	92.02		5.9	5.6	7.8
Pipemidic acid	5	114.1	100.5	91.28		7.7	9.4	3.5
	50	94.10	83.39	88.21		8.7	6.4	7.4
Danofloxacin	5	83.69	90.52	75.61		9.4	4.9	6.8
	50	75.51	86.03	81.89		5.9	7.2	7.0
Oxolinic acid	5	102.7	84.29	83.47		9.4	6.3	8.5
	50	85.82	71.83	73.68		9.1	6.3	7.0
Enrofloxacin	5	106.4	111.8	87.26		8.4	5.5	7.4
	50	75.73	89.95	95.11		6.6	5.3	6.7
Compound	Spiked/ (μg/kg)	Mean recoveries/%				RSDs/%		
		Rice powder	Meat paste	Fish paste		Rice powder	Meat paste	Fish paste
Sulfadoxin	5	84.01	116.8	111.3		7.4	10.3	9.9
	50	92.79	95.47	107.1		13.0	8.4	7.6
Sulfachloropyridazine	5	73.95	117.8	113.8		11.5	10.5	10.0
	50	95.65	71.25	110.7		6.1	9.1	9.2
Sulfadiazine	5	95.94	74.07	108.7		8.2	15.4	12.7
	50	81.12	99.27	101.8		11.2	9.0	10.2
Sulfathiazole	5	101.4	72.91	112.7		5.7	11.7	10.8
	50	88.96	106.7	115.6		11.7	9.4	10.0
Erythromycin	5	73.91	75.24	70.94		7.5	7.2	7.5
	50	76.99	79.09	93.23		5.0	6.2	6.8
Tilmicosin	5	81.95	69.36	65.62		10.0	7.9	8.1
	50	101.3	74.52	105.1		3.9	6.0	6.2
Metronidazole	5	71.70	101.2	87.15		9.7	8.7	8.3
	50	103.3	97.19	92.18		11.6	10.1	10.0
Metronidazole-OH	5	100.9	93.29	97.64		12.1	6.2	7.3
	50	112.8	91.75	90.04		1.1	6.7	7.0
Dimetridazole	5	93.93	100.1	86.08		4.0	8.3	8.0
	50	78.25	93.13	88.35		10.8	5.2	5.9
Hydroxydimetridazole	5	88.88	104.7	94.76		12.1	6.6	6.5
	50	100.1	78.73	89.15		9.5	8.7	8.7
Ronidazole	5	78.79	84.58	86.26		9.0	7.2	6.9
	50	104.8	81.04	78.18		9.2	8.8	8.4
Ipronidazole	5	97.68	104.4	87.36		6.3	9.5	8.8
	50	76.53	73.83	83.44		8.4	7.0	5.0
Ipronidazole-OH	5	98.43	93.35	94.63		11.5	13.2	10.5
	50	89.16	88.68	97.34		1.6	9.9	11.9
Amantadine	5	96.22	106.5	90.41		13.1	9.1	8.6
	50	72.36	115.3	83.61		5.6	9.2	12.7
Rimantadine	5	92.25	110.8	85.47		8.7	9.7	10.8
	50	80.56	92.11	93.45		12.5	8.9	9.9
Florfenicol amine	5	108.7	85.03	93.02		12.7	10.3	9.5
	50	109.6	66.41	66.53		13.6	9.7	11.9
Chloramphenicol	5	112.1	82.14	103.1		4.4	8.9	7.7
	50	95.89	91.09	88.53		3.8	5.0	7.6
Florfenicol	5	94.01	93.55	88.88		4.9	9.5	9.6
	50	79.85	69.99	76.68		4.3	4.3	6.9
Thiamphenicol	5	112.3	104.2	86.17		9.0	11.8	11.5
	50	89.43	92.46	83.77		8.1	7.9	6.2
Compound	Spiked/ (μg/kg)	Mean recoveries/%				RSDs/%		
		Rice powder	Meat paste	Fish paste		Rice powder	Meat paste	Fish paste
Flumequine	5	100.4	104.1	92.64		9.1	7.1	7.5
	50	94.42	75.04	88.41		6.8	6.4	6.7
Fleroxacin	5	100.9	104.2	86.79		7.4	8.7	6.0
	50	74.37	77.57	87.49		3.0	5.0	6.7
Ciprofloxacin	5	89.67	96.55	72.07		8.5	4.8	5.8
	50	77.78	64.46	82.74		6.1	3.4	5.3
Lomefloxacin	5	76.96	106.4	93.43		8.2	9.1	6.7
	50	89.95	82.33	100.4		7.2	5.9	6.9
Marbofloxacin	5	100.7	97.42	83.69		5.7	6.8	7.9
	50	78.06	89.07	74.11		8.7	3.2	3.9
Nalidixic acid	5	110.8	101.9	104.7		7.5	12.8	13.2
	50	91.44	75.97	85.73		5.0	2.9	3.1
Norfloxacin	5	107.1	117.0	87.09		9.9	12.7	11.2
	50	73.39	68.92	90.16		3.9	3.8	3.8
Pefloxacin	5	81.53	88.48	78.17		9.7	2.7	3.3
	50	79.95	77.69	79.05		5.6	3.1	4.2
Sarafloxacin	5	87.51	96.72	89.64		2.1	2.7	3.6
	50	80.33	74.90	80.27		1.1	3.0	3.5
Difloxacin	5	110.4	92.96	89.36		4.0	2.8	2.8
	50	104.0	68.44	73.34		9.8	2.2	2.5
Sparfloxacin	5	84.06	109.2	85.54		8.1	2.3	2.0
	50	106.2	96.94	102.5		9.5	2.7	3.1
Cinoxacin	5	104.8	80.66	107.9		9.0	1.4	1.5
	50	114.8	94.21	83.74		9.2	2.7	3.0
Ofloxacin	5	96.53	97.44	77.18		6.3	2.2	3.4
	50	78.29	94.11	89.93		8.4	3.7	5.4
Enoxacin	5	109.3	83.36	91.15		4.5	7.7	6.4
	50	79.56	64.57	64.37		6.6	4.2	5.9
Sulfapyridine	5	93.52	81.84	104.2		13.1	9.8	13.8
	50	70.60	100.4	104.9		5.6	6.5	6.8
Sulfameter	5	79.39	82.05	100.5		8.7	6.6	8.8
	50	86.36	108.1	115.2		5.5	5.9	6.6
Sulfamethazine	5	75.18	86.67	93.64		6.7	6.7	8.2
	50	72.79	113.6	119.3		8.6	6.4	9.0
Sulfisoxazole	5	106.0	68.55	114.4		3.8	5.8	8.1
	50	83.93	86.66	110.6		4.9	4.2	7.0
Sulfisomidine	5	91.08	114.3	98.13		4.4	5.3	5.9
	50	76.24	95.98	112.1		9.3	8.6	7.4
Sulfamethoxazole	5	114.7	69.31	110.3		9.0	7.9	4.6
	50	92.69	69.54	107.2		8.1	8.0	5.5
Sulfamerazine	5	106.6	71.07	106.1		13.2	8.3	7.1
	50	74.54	112.1	98.37		5.9	11.0	11.4
Sulfamethizole	5	95.27	98.16	109.6		7.7	14.4	12.5
	50	98.85	78.12	118.8		10.0	9.8	9.8
Sulfamethoxypyridazine	5	74.90	83.97	97.16		9.4	9.7	8.7
	50	86.92	96.46	105.3		5.9	8.8	11.2
Sulfadimethoxine	5	84.69	71.66	107.6		9.4	10.6	9.5
	50	83.72	68.66	79.74		9.1	9.9	10.6
Sulfamonomethoxine	5	87.14	114.8	100.5		8.4	8.4	9.1
	50	94.13	113.2	117.3		10.6	11.4	11.6
Sulfaquinoxalin	5	89.66	115.8	106.4		9.1	13.5	12.4
	50	92.98	68.87	83.51		13.8	8.7	8.1

### 2.4 实际样品的检测

采用本方法对市面上购买的20份婴幼儿辅助食品(米粉7份、肉泥7份、鱼泥6份)进行检测,其中3份肉泥和3份鱼泥为从网上购买的进口产品,其余产品均为国内生产。

定量分析采用的基质标准工作溶液,以成分相近的阴性样品制备所得的空白基质溶液来配制。虽然定量分析采用的相近基质可能无法与待测样品的基质性质完全匹配,但是样品中兽药残留含量的测定结果也存在一定的参考意义。而且本方法检出限较低,灵敏度高,采用成分相近的基质匹配标准曲线定量,不会造成假阳性或假阴性的结果。

检测结果显示,国内生产的婴幼儿辅助食品中未检测出本方法中的50种兽药残留。然而,一个进口的肉泥样品中检测出了磺胺喹噁啉、磺胺二甲嘧啶和替米考星,检出值分别为8.2、10.3、15.8 μg/kg。由此可见,婴幼儿辅助食品的兽药残留监管应得到更多的重视,尤其是针对进口的婴幼儿辅助食品。

为进一步验证方法的准确性和可行性,针对此肉泥检出的兽药,选择适用范围较合适的现行标准方法进行检测,其中,磺胺喹噁啉和磺胺二甲嘧啶采用农业部1025号公告-23-2008^[26]^方法测定,替米考星采用GB/T 20762-2006^[27]^方法测定。结果显示,磺胺喹噁啉、磺胺二甲嘧啶和替米考星的检出值分别为7.9、11.0、15.1 μg/kg,与本方法得到的结果基本一致,表明本方法准确可靠。

## 3 结论

本工作采用酸化乙腈超声提取样品,提取液经脂质增强型固相萃取柱净化,然后经超高效液相色谱-三重四极杆质谱仪检测,建立了婴幼儿辅助食品中50种兽药残留的检测方法。该方法下50种兽药均具有较低的检出限和定量限,且回收率较高,重复性好。本方法前处理简单快速,没有繁琐的步骤,经济可靠,准确度和灵敏度高,样品量消耗少,适用于婴幼儿辅助食品中多兽药残留的定性定量分析。
